# Hyperglycemia in Hospitalized Patients Receiving Parental Nutrition Is Associated with Increased Morbidity and Mortality: A Review

**DOI:** 10.1155/2011/760720

**Published:** 2010-08-03

**Authors:** Puja Rajender Kumar, Pam Crotty, Maitreyi Raman

**Affiliations:** Division of Gastroenterology, Department of Medicine, University of Calgary, 6D26, Teaching, Research and Wellness Building, 3280 Hospital Drive N.W., Calgary, AB, Canada T2N 4N1

## Abstract

Parenteral Nutrition (PN) is a valuable life saving intervention which can improve the nutritional status of hospitalized malnourished patients. PN is associated with complications including the development of hyperglycemia. This paper aims to provide a descriptive systematic review regarding the effects of PN-induced hyperglycemia in hospitalized patients, either in the intensive care unit or ward, while formulating and complementing existing guidelines on the administration of PN and glucose monitoring in hospitalized patients. Medline and Pubmed were searched for relevant articles describing complications arising from the development of hyperglycemia in patients receiving PN; four relevant studies were identified in the search. These articles had different glycemic targets and patient populations, and their protocols varied with regards to glycemic control. However, there was consistency regarding the association between hyperglycemia and mortality in patients receiving PN. These studies highlight the need for guidelines regarding monitoring and initiation of therapy in hyperglycemic patients. Unfortunately, all the currently available studies are retrospective in design; a large, prospective, randomized controlled trial regarding glycemic control in patients receiving PN is required for the development of standardized protocols.

## 1. Introduction

Parenteral nutrition (PN) is a form of intravenous nutritional support, originally developed at the University of Pennsylvania School of Medicine in 1968 to support malnourished surgical patients [1]. Shortly thereafter, PN was shown to be valuable in providing life-saving nutrition for both complex medical and surgical patients with a nonfunctioning GI tract. It has been well established that PN has a beneficial effect in improving the nutritional status of hospitalized malnourished patients [2] and is predominately used in those patients who are unable to receive nutrition either orally or enterally largely due to intestinal failure. Despite the life saving benefits attributed to PN, it is known to be associated with a number of short- and long-term complications including liver disease, catheter-related sepsis, septic shock, fluid and electrolyte abnormalities, and hyperglycemia. Arguably, the interest in blood glucose control and subsequent consequences of hyperglycemia among hospitalized patients receiving PN is rapidly increasing, mirroring the interest in the general inpatient population. The mechanism of harm from hyperglycemia on various organ systems has not been well defined but it is known that hyperglycemia alters the activity of phagocytes, interfering with neutrophil and monocyte functions [3]. Hyperglycemia also increases inflammatory cytokines, oxidative stress and promotes apoptosis [4–7]. Cell and tissue injury caused by hyperglycemia through oxidative stress adversely affects the immune, cardiovascular and nervous system as well as hemostasis, inflammation, and endothelial cell function [8]. 

Recent groups have described an increase in medical complications and mortality occurring in both critically ill and noncritically ill hyperglycemic inpatients receiving PN [9–12]. The prevalence of hyperglycemia occurring in patients receiving PN is quite variable and ranges between 10–88% [9, 13, 14]. It has been well established that in- hospital hyperglycemia occurring in patients without any attributable risk factor is associated with higher mortality rates. [15–17]. Three recent studies of hospitalized patients including both critically ill and noncritically ill, identified PN-associated hyperglycemia as a risk factor for development of infection, cardiac, and renal dysfunction and increased mortality [9–11]. A fourth study of hospitalized, noncritically ill patients receiving PN found hyperglycemia to be a risk factor for increased mortality alone [12]. Therefore, the purpose of this manuscript is to provide a descriptive systematic review examining the medical complications of parenteral nutrition-associated hyperglycemia, and its association with mortality in critically ill and noncritically ill patients. It will also review glucose monitoring and therapy regimens and its effect in hospitalized inpatients.

## 2. Methods

A systematic review was carried out by two reviewers to search for articles relevant to this topic using Medline and PubMed applying the following search terms alone and in combination: hyperglycemia, total parental nutrition, and hospitalized patients. There were no language or time frame limits. Inclusion criteria were articles that examined hospitalized patients, critically or noncritically ill, receiving parenteral nutrition and the effects of hyperglycemia on this population compared with those who did not develop hyperglycemia. Articles were excluded if they did not specifically look at this population. However, clinical studies, reviews, consensus statements, and meta-analysis relevant to the identification and management of hospitalized hyperglycemic patients receiving PN were selected and included. Articles found were assessed for eligibility and compared between the two reviewers. The references and citations were also reviewed to identify other relevant articles. Data extracted included patient demographics, mean glucose level, study definition of hyperglycemia, method of glucose monitoring, duration of PN, outcomes/complications associated with the development of hyperglycemia while receiving PN (i.e., mortality, acute renal failure, any complication, any infection, etc.) as well as their odds ratios and 95% confidence intervals. Authors were contacted via email if the papers lacked data that pertained to this study. The studies were heterogeneous in their methods, therefore the data could not be combined statistically, but general trends were assessed.

## 3. Results

The search resulted in 38 possible articles, which were further narrowed down to four articles that met our eligibility criteria. There was a kappa agreement of 100% between the two reviewers on the inclusion of these articles. These four retrospective studies [9–12] explored the relationship between hyperglycemia and health outcomes and are described in Table 1. Demographics were underreported in these studies and hence they could not be combined for this review. 

Cheung et al. [9] were the first group to look at adverse outcomes associated with PN-induced hyperglycemia. They conducted a retrospective analysis reviewing 109 hospitalized patients in the ward or the intensive care unit, who received PN during the year 2002 in the Westmead Hospital, Sydney, Australia. Mean blood glucose levels were calculated from daily serum glucose readings taken for the duration that patients were receiving PN. Hyperglycemic patients, defined as having blood glucose greater than 10 mmol/L, would undergo blood glucose testing q4 hours by finger prick and PN calories would be reduced. If the blood glucose level remained greater than 10 mmol/L, the protocol called for commencement of an insulin infusion for the duration of PN therapy irrespective of whether the patient was critically ill or not. Outcome measures included development of any infection (culture proven), septicemia (blood culture proven), cardiac complication (myocardial infarction, cardiac arrhythmia, and cardiac arrest), acute renal failure, or death (during admission). The mean duration of PN was 12.1 ± 20.4 days and the mean daily blood glucose during PN was 8.0 ± 1.5 mmol/L.

Lin et al [10] conducted a similar study in a group of patients, also with mixed indications, in the Taipei Veterans General Hospital, Taipei, Taiwan during the year 2004. A retrospective cohort study including 457 hospitalized patients was undertaken to determine associations between hyperglycemia and adverse outcomes in patients receiving PN. All euglycemic subjects had serum glucose measurements twice a week. In this study, patients were defined as having hyperglycemia if a single blood glucose measurement was greater than 6.3 mmol/L. Hyperglycemic patients underwent capillary glucose testing q6 hours by finger prick. The number of blood glucose values per patient ranged from 1 to 207; the mean blood glucose level was calculated using these capillary glucose readings. Treatment of hyperglycemia was not outlined in the methods. Outcome measures included parameters described by Cheung et al. [9]. Additionally, documented bacteremia, fungemia, and respiratory failure defined as the requirements for mechanical ventilation while receiving PN were included as additional clinical outcomes. The mean duration of PN was 17.8 ± 17.7 days and the mean daily blood glucose was 8.6 ± 3.2 mmol/L. 

Pasquel et al. [11] conducted a retrospective study of 276 hospitalized patients, in the ward and the intensive care unit at the Grady Memorial Hospital, Atlanta, Georgia, United States during the year 2006. Blood glucose levels on admission, pre-PN, within 24 hours of initiation of PN, and during days 2–10 of PN were included in the analysis. Hyperglycemia was defined as a blood glucose level above 6.7 mmol/L. The monitoring and treatment of these patients was not outlined in the methodology. Outcome measures included mortality, development of any infections, length of stay (LOS), and renal failure. The mean duration of PN was 15 ± 24 days. The mean daily blood glucose on admission was 7.7 ± 4.7 mmol/L. The mean blood glucose prior to initiation of PN was 6.8 ± 1.8 mmol/L and increased to a mean blood glucose of 8.1 ± 2.4 mmol/L within 24 hours and remained elevated at 7.8 ± 2.2 mmol/L for the remainder of the days analyzed. 

Sarkisian et al. [12] conducted a retrospective study of 100 hospitalized patients in the Foothills Medical Centre, Calgary, Canada. This cohort excluded critically ill individuals unlike the preceding three studies outlined [9–11]. Mean blood glucose values were calculated for each patient based on the total number of readings during their first 9 days receiving PN including serum and finger prick readings. Hyperglycemia was defined as mean blood glucose levels above 10mmol/L. Outcomes measured included development of any infection, acute coronary event, acute renal failure, LOS, ventilator use, ICU admission, and death. Additionally, this group reviewed the median frequency of glucose monitoring in both euglycemic and hyperglycemic patients and the association of the frequency of monitoring with mortality. 

### 3.1. Mortality

All four studies showed significantly increased mortality in patients with mean blood sugars greater than 10 mmol/L while receiving PN after adjusting for age, gender, and previous diabetes status, when compared to a lower blood glucose group (Table 2). Pasquel et al. [11] were the only group to examine blood glucose levels at differing time periods in relation to PN initiation. They grouped the patients into three groups; pre-PN initiation, within 24 hours of PN initiation, and during days 2–10 of PN. In comparison to living patients, deceased patients in their cohort had a significantly higher blood glucose pre-PN (7.2 ± 2.1 mmol/L versus 6.7 ± 1.8 mmol/L), within 24 hours of PN initiation (9.0 ± 3.1 mmol/L versus 7.7 ± 2.1 mmol/L) and during days 2-10 of PN (8.9 ± 2.9 mmol/L versus 7.9 ± 1.9 mmol/L). Their study indicates that blood glucose values prior to and within 24 hours of initiation of PN are better predictors of hospital mortality and complications than the mean blood glucose during the entire duration of PN. Pasquel et al. found that mortality was independently predicted by pre-PN blood glucose values between 8.4-10mmol/L (OR 3.41, 95% CI 1.3–8.7, *P* < .01) and greater than 10mmol/L (OR 2.2, 95% CI 0.9–5.2, *P* = .077), as well as by blood glucose within 24 hours of PN greater than 10 mmol/L (OR 2.8 95% CI 1.2–6.8, *P* = .020) versus patients without hyperglycemia.

### 3.2. Complications

Not all the groups agreed on the association between hyperglycemia and complications (Table 2). Sarkisian et al. [12] did not find an association between hyperglycemia and acute coronary events, renal failure, infection, hospital length of stay, ventilator use, or admission to a critical care unit. This may be in part due to the less critically ill population studied in comparison to the other three studies which included both critically ill and noncritically ill patients. Cheung et al. [9] found that for every 1 mmol/L increase in blood glucose above 6.9 mmol/L, the risk of any complication increased by a factor of 1.58. Patients with a mean blood glucose greater than 9.1 mmol/L had the highest relative risk for complications (OR of 4.3, 95% CI 1.4–13.1 *P* = .01) using blood glucose of less than 6.9 mmol/L as the reference. Lin et al. [10] found a similar association. For every 0.56 mmol/L increase in blood glucose above 6.3 mmol/L the risk of any complication increased by a factor of 1.14. The highest relative risk of complications was in the group of patients with mean blood glucose greater than 10 mmol/L (OR of 5.5, 95% CI 2.5–12.4 *P* < .001) using blood glucose of less than 6.3 mmol/L as the reference category. Pasquel et al. [11] noted that patients with higher blood glucose levels during TPN had a longer hospital (*P* = .011) and ICU length of stay (*P* = .008). They also found an association between the risk of pneumonia (OR=3.6, 95% CI 1.6–8.4) and acute renal failure (OR=2.2, 95% CI 1.0–4.8) in patients with blood glucose greater than 10 mmol/L during the first 24 hours of TPN compared with patients having a mean blood glucose less than 6.7 mmol/L.

### 3.3. Monitoring

Blood glucose monitoring methods and frequency was reported in each of the studies' methods, but not all examined the effect on outcomes. Unfortunately, it was not reported if monitoring was considerably different between the critically ill and noncritically ill patients included in these studies. Of these four studies, only Sarkisian et al. [12] examined the frequency of blood glucose monitoring and outcomes related to this. Patients with hyperglycemia had their blood glucose monitored more frequently in both the first 48 hours (median 4 times, IQR: 2–7, *P* = .003) and in the first week (median 6 times, IQR: 3–18, *P* ≤ .001) compared to euglycemic patients. There was no association between frequency of glucose monitoring and mortality in the first 48 hours of PN or in the subsequent week of PN. The other studies reported varying schedules to monitor hyperglycemia and did not statistically analyze complications or mortality associated with monitoring frequency.

## 4. Discussion

In our descriptive systematic review of the four available retrospective studies examining hyperglycemia in hospitalized patients receiving PN, one consistent finding was observed; mortality was increased significantly if blood sugars were above 10 mmol/L. Unfortunately, of the published studies examining hyperglycemia in PN patients, glycemic targets differed, patient populations were not identical, protocols for monitoring blood sugars varied, and there was a lack of information regarding hyperglycemic and euglycemic control. These heterogeneous methods likely account for the variations in results regarding complications and morbidity associated with hyperglycemia. Three of these studies included both critically ill and noncritically ill patients and assessed outcomes in a homogeneous manner, not accounting for potential confounding factors in their analysis such as the indication for PN. This limitation in study design demonstrates the need for the establishment of large, controlled trials regarding glycemic control in more homogenous patients receiving PN, either critically ill or noncritically ill, for the development of standardized protocols regarding monitoring and glucose therapy in PN patients.

All four studies failed to monitor blood glucose to the level suggested by The American Society of Parenteral and Enteral Nutrition (A.S.P.E.N) [18]. To our knowledge, this expert organization in nutrition support practices has the only published set of established guidelines regarding glucose monitoring in patients receiving PN. They suggest glucose monitoring q6 hours upon initiation of PN and at least three times daily within days 3–9 until the blood glucose has reached less than 11mmol/L. These guidelines do not give further recommendations regarding closer monitoring for critically ill patients. Based on the findings from Cheung, Lin, Pasquel, and Sarkisian [9–12], the level of 11mmol/L recommended by A.S.P.E.N as an indicator of acceptable blood sugars may need to be further reduced. 

Several studies with heterogeneous designs and outcome measures have examined the relationship between tight glycemic control and outcomes in critically ill patients. Griesdale and colleagues report results from a meta-analysis of 26 studies involving over 13,500 patients [19]. The original landmark study conducted by Van den Berge et al. [20] compared intensive insulin therapy versus conventional treatment among surgical intensive care patients, predominantly PN fed. Fasting blood glucose targets were 4.4–6.1 mmol/L and 10-11.1 mmol/L in the intensive and conventional arms, respectively. They demonstrated a 34% decrease in mortality with intensive insulin therapy. However, subsequent studies in slightly different populations have failed to show such benefit. The Normoglycemia in Intensive Care Evaluation-Survival Using Glucose Algorithm Regulation (NICE-SUGAR) is currently the largest randomized controlled study comparing intensive versus conventional glucose control among both surgical and medical intensive care patients who were predominantly enterally fed. The NICE-SUGAR study defined intensive glucose control with a target blood glucose range of 4.5–6.0 mmol/L and conventional control as a target of 10.0 mmol/L or less. The authors found that intensive glucose control increased the absolute risk of death at 90 days by 2.6% compared with conventional glucose control. This represents a number needed to harm of 38. There was also a 6-fold increase in the rate of occurrence of hypoglycemia with use of intensive therapy in all ICU patients [21]. Since the publication of these studies and the recent meta-analysis, the American College of Endocrinology (ACE) and the American Diabetes Association (ADA) have generated a consensus statement which adopts less stringent blood glucose targets between 7.8–10.0 mmol/L for critically ill patients to prevent hypoglycemia, while controlling for hyperglycemia [22, 23]. They also recommend intravenous insulin infusions for critically ill patients and subcutaneous basal-bolus, prandial and correctional dosing for all noncritically ill patients [24, 25]. Based on the aggregated results from the 4 studies examining outcomes in PN patients and the guidelines from the ACE and the ADA described above, we could conclude that the mean blood glucose in patients receiving PN, whether they are critically ill or noncritically ill, should be less than 10 mmol/L and potentially the appropriate target range would lie between 6.3 to 9.1 mmol/L. 

Only Cheung et al. clearly outlined treatment of hyperglycemia in their patients. Their protocol called for an insulin infusion for all patients irrespective of their inpatient location if during PN treatment blood sugars were persistently above 10 mmol/L. Treatment of hyperglycemia with insulin infusion was associated with an increased rate of complications (OR 2.7, 95% CI 1.3–5.6, *P* = 0.01), although not statistically significant for death. There were no documented cases of hypoglycemia in patients receiving insulin infusions. Incidentally, the mean blood glucose levels for these patients were significantly higher than patients who did not require insulin therapy, suggesting that insulin infusions were inadequately administered. This was the only study in our review to examine treatment of hyperglycemia, and the evidence is inconclusive regarding harm or benefit of the use of insulin infusions in patients receiving PN, furthering the need for more evidence regarding treatment of hyperglycemia in PN patients.

Currently, as recommended by ACE/ADA, insulin is the most appropriate agent for management of hyperglycemia [25]. There are many methods by which insulin can be administered to patients receiving PN, including subcutaneous administration, insulin infusion, addition of insulin to the PN bag, or a combination of these methods. No head to head comparisons of these methods are available at this time to comment on the best available technique. Previously, there was a controversy surrounding the amount of insulin available when mixed with PN solutions. However, Dunham et al. [26] published a very well-designed study showing the recovery of regular insulin from all-in-one PN mixtures in ethylene vinyl acetate bags to be up to 95%. Surprisingly, there have been very few studies describing glucose control in PN patients with hyperglycemia treated with insulin provided in the PN mixture. There is some discordant opinion among PN experts regarding insulin delivery via this method, and therefore no consistent comment can be provided regarding the safety and effect of this method of intervention. 

Glucose management strategies in the critically ill PN recipient should involve frequent glucose monitoring and implementation of IV insulin infusion. The 2009 ACE/ADA recommends IV insulin infusions for all critically ill patients. The target blood glucose is recommended to be kept between 7.8 to 10 mmol/L. Administration of insulin infusions to PN patients with hyperglycemia in the noncritically-ill setting would be a significant burden on the system, given the extra monitoring required and nursing care involved. In the ICU setting, one-on-one nursing allows the adoption of a tighter glycemic control/insulin protocols; however, this is not practical for ward patients. 

Glucose management strategies in the noncritically ill PN recipient typically involve subcutaneous insulin in a variety of breakdowns. The ACE/ADA recommends basal/bolus/correction subcutaneous insulin regiments tailored to the individual patient with adequate adjustments as the patient condition changes, Targeting random blood glucose levels of less than 10 mmol/L, and once the patient is eating premeal blood glucose targets of less than 7.8 mmol/L. A multidisciplinary approach may be necessary for successful implementation of effective glycemic management in the hospital setting. Individualized plans taking into account the patient profile, nursing support, and monitoring capabilities are likely the most prudent way to proceed until further studies are done regarding optimal therapy regimens. Most noncritically ill patients can be managed effectively using subcutaneous route of delivery. However, sliding scale regimens, defined as administration of a preestablished amount of rapid acting insulin in response to hyperglycemia, are ineffective [23, 27]. This method does not emulate the natural circadian rhythm; it is more reactive than proactive and does not include basal insulin. The ACE/ADA discourages use of these sliding scales due to wide fluctuations in glucose levels documented in several observational studies. A prospective randomized in 130 hospitalized noncritically ill patients with type 2 diabetes showed that basal-bolus insulin regiment (using a daily long-acting insulin analog with preprandial rapid-acting insulin analog) was superior to a standard sliding scale protocol. The target blood glucose of less than 7.8 mmol/L was achieved in 66% of patients in the basal-bolus group versus only 38% of patient in the sliding scale group [28].

Management of hyperglycemia and insulin dosing in patients receiving parenteral nutrition should be done on an individual basis, regardless of indication. Insulin dosing requirements may change rapidly as the patient's underlying illness evolves. It is recommended that insulin requirements be reassessed after any changes in nutritional status, and certainly with changes in the nutrition support prescription and with changes in oral intake. Close glucose monitoring with frequent insulin dose adjustments is a critical part of effective glycemic management, and a generalized cookbook approach may not be suitable for patients on PN. The A.S.P.E.N guidelines recommend q6 hour capillary blood sugars. All four studies had less than optimal glucose monitoring in their hyperglycemic patients and only one looked at outcomes associated with monitoring. Sarkisian et al. [12] did not show an increased mortality associated with frequency of glucose monitoring. However, this was a retrospective study including only 100 patients in a noncritically ill setting.

We can only infer a trend towards increased mortality and morbidity in hyperglycemic PN patients from these studies because they were limited in their design; all were retrospective and differed in glycemic targets. Their patient populations were not identical and their protocols for monitoring blood sugars varied. It is difficult to conclude from these studies that hyperglycemia alone led to the observed complications and increased mortality, as preexisting comorbid conditions and indications for PN were not accounted for. Additionally, as we could not separate the critically ill from the noncritically ill in these studies, the increased mortality seen in patients with poorly controlled glucose may have been a result of preexisting conditions and a sicker population. Our review has inherent limitations since our search yielded only four papers which varied in their design not allowing us to statistically combine the results or draw sound conclusions.

## 5. Conclusion

Hyperglycemia is associated with poor outcomes in patients receiving PN; this applies to patients with and without diabetes and both critically ill and noncritically ill. There is a significant increase in mortality when blood sugars are above 10 mmol/L. There is a suggestion of increased complication rates in this patient population but further studies need to be done to confirm these findings. These studies suggest that the most acceptable level of blood glucose should range between 6.3–9.1 mmol/L. Conclusions regarding the most appropriate method for management of hyperglycemia could not be drawn from this cohort due to lack of information regarding therapy. Given the lack of standardized protocols regarding monitoring and therapy in this population, a large prospective, randomized controlled trial in patients receiving PN with hyperglycemia is necessary to determine optimal monitoring, optimal delivery of insulin, and whether the control of blood glucose can improve outcomes in this patient population.

## Figures and Tables

**Table 1:** Characteristics of the studies examining hyperglycemia in patients receiving PN.

Study	Cheung (2005)	Lin (2007)	Sarkisian (2009)	Pasquel (2009)
Study Design	Retrospective	Retrospective	Retrospective	Retrospective
^#^of Patients	109	457	100	276
Patient Population	Mixed	Mixed	Mixed	Noncritically Ill only
Mean Age ± SD	51.9 ± 18.7	66.4 ± 16.3	61.9 ± 17	51 ± 18
Glucose Monitoring	Daily and capillary glucose testing q4 hours for duration of PN	Twice weekly and capillary glucose testing q6 hours for duration of PN	Variable, including first 9 days on PN	On admission, pre PN and daily for PN day 1–10
Mean Glucose	Blood Draw	Finger Prick	Blood Draw & Finger Prick	Blood Draw
Blood glucose cut points examined (mmol/L)	<6.9	<6.3	<10	<6.7
	6.9−7.8	6.3−7.6	≥10.0	6.7−8.3
	7.9−9.1	7.6−10		8.4−10
	>9.1	>10.0		>10.0

**Table 2:** Risk of mortality and complications due to hyperglycemia in patients receiving PN.

Study	Cheung (2005)	Lin (2007)	Sarkisian (2009)	Pasquel (2009)
Hyperglycemia(mmol/L)	>9.1*	>10**	>10***	>10****
Mortality OR(95%CI)	10.90(2.0 − 60.5)^X^	5.0(2.4 − 10.6)^X^	7.22(1.08 − 48.3)^X^	2.80(1.20 − 6.80)^X^
Any Infection OR(95%CI)	3.9(1.2 − 12.0)^X^	3.1(1.5 − 6.5)^X^	0.9(0.3−2.5)	NA
Cardiac OR(95%CI)	6.2(0.7−57.8)	1.6(0.3-7.2)	1.3(0.1−12.5)	NA
Acute Renal Failure OR(95%CI)	10.9(1.2 − 98.1)^X^	3.0(1.2 − 7.7)^X^	1.9(0.4−8.6)	2.2(1.0−4.8)
Septicemia OR(95%CI)	2.5(0.7−9.3)	NA	NA	NA
Any Complication OR(95%CI)	4.3(1.4 − 13.1)^X^	5.5(2.5 − 12.4)^X^	NA	NA

All study results are adjusted for age and sex.

^X^ Significant at *P* < .05.

*ORs are expressed using blood glucose <6.9 mmol/L as a reference category.

**ORs are expressed using blood glucose <6.3 mmol/L as a reference category.

***ORs are expressed using blood glucose <10 mmol/L as a reference category.

****ORs are expressed using blood glucose <6.7 mmol/L as a reference category as measured within 24 hours of PN initiation.

## References

[1] Brokenshire E, Plank LD, Gillanders LK, McIlroy K, Parry BR (2009). Adult total parenteral nutrition at Auckland City Hospital: a 6-year review. *The New Zealand Medical Journal*.

[2] Ziegler TR (2009). Parenteral nutrition in the critically ill patient. *The New England Journal of Medicine*.

[3] Leibovici L, Yehezkelli Y, Porter A, Regev A, Krauze I, Harell D (1996). Influence of diabetes mellitus and glycaemic control on the characteristics and outcome of common infections. *Diabetic Medicine*.

[4] Esposito K, Nappo F, Marfella R (2002). Inflammatory cytokine concentrations are acutely increased by hyperglycemia in humans. *Circulation*.

[5] Marfella R, Nappo F, De Angelis L, Paolisso G, Tagliamonte MR, Giugliano D (2000). Hemodynamic effects of acute hyperglycemia in type 2 diabetic patients. *Diabetes Care*.

[6] Wong V, Ross DL, Park K, Boyages S, Cheung NW (2004). Hyperglycemia following acute myocardial infarction is a predictor of poor cardiac outcomes in the reperfusion era. *Diabetes Research and Clinical Practice*.

[7] Ceriello A, Quagliaro L, D’Amico M (2002). Acute hyperglycemia induces nitrotyrosine formation and apoptosis in perfused heart from rat. *Diabetes*.

[8] Sheetz MJ, King GL (2002). Molecular understanding of hyperglycemia’s adverse effects for diabetic complications. *Journal of the American Medical Association*.

[9] Cheung NW, Napier B, Zaccaria C, Fletcher JP (2005). Hyperglycemia is associated with adverse outcomes in patients receiving total parenteral nutrition. *Diabetes Care*.

[10] Lin L-Y, Lin H-C, Lee P-C, Ma W-Y, Lin H-D (2007). Hyperglycemia correlates with outcomes in patients receiving total parenteral nutrition. *American Journal of the Medical Sciences*.

[11] Pasquel FJ, Spiegelman R, McCauley M Hyperglycemia during total parenteral nutrition (TPN): an important marker of poor outcome and mortality in hospitalized patients.

[12] Sarkisian S, Fenton T, Shaheen AA, Raman M Parenteral nutrition hyperglycemia in non-critically Ill inpatients is associated with higher mortality.

[13] van der Voort PHJ, Feenstra RA, Bakker AJ, De Heide L, Boerma EC, van der Horst ICC (2006). Intravenous glucose intake independently related to intensive care unit and hospital mortality: an argument for glucose toxicity in critically ill patients. *Clinical Endocrinology*.

[14] Schloerb PR (2004). Glucose in parenteral nutrition: a survey of US medical centers. *Journal of Parenteral and Enteral Nutrition*.

[15] Capes SE, Hunt D, Malmberg K (2000). Stress hyperglycaemia and increased risk of death after myocardial infarction in patients with and without diabetes: a systematic overview. *Lancet*.

[16] Capes SE, Hunt D, Malmberg K (2001). Stress hyperglycaemia and increased risk of death after myocardial infarction in patients with and without diabetes: a systematic overview. *Stroke*.

[17] Baker EH, Janaway CH, Philips BJ (2006). Hyperglycaemia is associated with poor outcomes in patients admitted to hospital with acute exacerbations of chronic obstructive pulmonary disease. *Thorax*.

[18] Sacks GS, Mayhew S, Peterson C, Merritt R (2009). Parenteral nutrition implementation and management. *ASPEN Nutrition Support Practice Manual*.

[19] Griesdale DEG, De Souza Rd RJ, van Dam RM (2009). Intensive insulin therapy and mortality among critically ill patients: a meta-analysis including NICE-SUGAR study data. *Canadian Medical Association Journal*.

[20] Van Den Berghe G, Wouters P, Weekers F (2001). Intensive insulin therapy in critically ill patients. *The New England Journal of Medicine*.

[21] Finfer S, Bellomi R, Blair D (2009). Intensive versus conventional glucose control in critically Ill patients. *New England Journal of Medicine*.

[22] Clark NG, Fonseca V, Garber AJ, Inzucchi SE, Moghissi ES (2006). American College of Endocrinology and American Diabetes Association consensus statement on inpatient diabetes and glycemic control. *Endocrine Practice*.

[23] American Diabetes Association (2009). Standards of medical care in diabetes 2009. *Diabetes Care*.

[24] Moghissi ES, Korytkowski MT, Dinardo M (2009). American Association of Clinical Endocrinologists and American Diabetes Association consensus statement on inpatient glycemic control. *Endocrine Practice*.

[25] Moghissi ES (2010). Addressing hyperglycemia from hospital admission to discharge. *Current Medical Research & Opinion*.

[26] Dunham B, Marcuard SP (1990). Availability of insulin from TPN solutions. *Journal of Parenteral and Enteral Nutrition*.

[27] Mullen BA, Watts Kelley PA (2006). Diabetes nurse case management: an effective tool. *Journal of the American Academy of Nurse Practitioners*.

[28] Umpierrez GE, Smiley D, Zisman A (2007). Randomized study of basal-bolus insulin therapy in the inpatient management of patients with type 2 diabetes (RABBIT 2 Trial). *Diabetes Care*.

